# Children with Physical Disabilities at School and Home: Physical Activity and Contextual Characteristics

**DOI:** 10.3390/ijerph14070687

**Published:** 2017-06-25

**Authors:** Ru Li, Cindy Hui-Ping Sit, Jane Jie Yu, Raymond Kim-Wai Sum, Stephen Heung-Sang Wong, Kenneth Chik-Chi Cheng, Thomas L. McKenzie

**Affiliations:** 1 Department of Sports Science and Physical Education, The Chinese University of Hong Kong, Hong Kong, China; lirutracy@link.cuhk.edu.hk (R.L.); yujie@cuhk.edu.hk (J.J.Y.); kwsum@cuhk.edu.hk (R.K.-W.S.); hsswong@cuhk.edu.hk (S.H.-S.W.); kchengc@cuhk.edu.hk (K.C.-C.C.); 2 School of Exercise and Nutritional Sciences, San Diego State University, San Diego, CA 92182, USA; tmckenzi@mail.sdsu.edu

**Keywords:** children, physical disability, physical activity, environmental correlates, observation

## Abstract

The purpose of this study was to assess the physical activity (PA) of children with physical disabilities (PD) in school and home settings and to simultaneously examine selected contextual characteristics in relation to PA in those settings. Children with PD (N = 35; Mean age = 15.67 ± 4.30 years; 26 boys) were systematically observed using BEACHES (Behaviors of Eating and Activity for Children’s Health: Evaluation System) at school (before school, recess, lunch break, after class) and at home (before dinner) during four normal school days. The children spent most of their time in all five settings being physically inactive, but had slightly more PA during recess and lunch break periods. Hierarchical multiple regression revealed that selected contextual characteristics explained 18.9–56.0% (*p* < 0.01) of the variance predicting moderate-to-vigorous physical activity (MVPA) after controlling for demographic variables. Prompts to be active were positively associated with MVPA at school and the presence of fathers and fathers being motivators at home. This study highlights how little PA that children with PD receive and identifies the importance of the provision of prompts for PA at both school and home with this special population.

## 1. Introduction

Regular physical activity (PA) in children with physical disabilities (PD) is important for their current and future health and well-being. Current health guidelines recommend that children should engage in 60 min or more of PA daily [1]. Evidence, however, indicates that children with PD are much less physically active than both their able-bodied counterparts [2] and their peers with other disability types [3,4]. They also tend to engage in fewer structured leisure time physical activities and to spend more time in sedentary pursuits [5]. This pattern of sedentary living puts children with PD at increased risk for decreased physical functioning [6], which could result in additional health problems that are associated with their primary disability [7]. To counter these increased health risks, there is a growing need for a better understanding of potentially modifiable variables that are associated with PA in this population.

The International Classification of Functioning, Disability, and Health (ICF) model recognizes the importance of environmental and personal factors that influence PA participation among persons with disabilities [8]. A recent review identified several physical, psychological, and environmental variables were positively associated with PA in children with PD [9]. During the past decade, public health has focused on the physical and built environment, resulting in a surge of studies on PA behavior and associated environmental attributes. Little emphasis, however, has been given specifically to children with disabilities. Previous reviews identifying correlates of PA among children with PD and environmental factors—such as activity area proximity and size, social support from family and peers, and the availability of equipment in home and school settings—greatly affected the extent and intensity of PA participation [10,11]. Although children with PD spend most of their time at school and home where they could accumulate PA, the physical and social contexts of these places (e.g., location, prompts from others) relative to PA have not been well documented.

While accelerometry has often been used to measure PA in children with PD [12], simultaneously capturing important aspects potentially influencing child’s PA is not always possible using this method. Meanwhile, direct observation methods exceed other measures of PA in their ability to provide detailed information on the physical and social contexts in which the activity occurs [13,14]. Identifying the characteristics of the environment in which PA occurs is well supported by both behavior analytic principles [15] and social ecological theory [16] and previous studies have used direct observation to study the PA of children with disabilities while simultaneously assessing contextual variables [4,17,18].

BEACHES (Behaviors of Eating and Activity for Children’s Health: Evaluation System) is a direct observation system that documents children’s PA and eating behaviors while simultaneously recording associated environmental characteristics and events in both home and school settings [19]. BEACHES has been validated and used with typically developing children [20,21,22], and accelerometry has been used to validate the BEACHES activity codes as suitable for assessing the PA of children with PD in both home and school settings [17]. In that study, children with PD were found to be more active during school recess than at home and their time spent outdoors was associated with more physical activity. The study was limited to assessing only five children with PD and, at school, only recess periods were examined; thus, our understanding of the PA children with PD in diverse school settings is limited.

Given that both homes and schools are important places where children can accrue PA, it is important to understand how active children with PD are in these settings and what factors may influence their activity levels. Therefore, the purpose of the present study was to (a) assess and compare the levels of PA of children with PD at home and school (recess, lunch break, and before and after school) settings and (b) examine the occurrence and association of selected environmental contextual variables with PA in those settings.

## 2. Materials and Methods

### 2.1. Participants

Participants were 35 Hong Kong Chinese children enrolled in grades 4 to 12 (Mean age = 15.67 ± 4.30 years) in a school designed especially to serve children with PD. Twenty-seven children were boarding students who lived in the school residential facility and eight children commuted from home daily. Only children who agreed to participate and had informed consent forms signed by their parents were included. Demographic and anthropometric data including body height and weight were obtained from the school, and Body Mass Index (BMI) was calculated (Mean BMI = 19.84 ± 4.84). The study was approved by the Joint Chinese University of Hong Kong-New Territories East Cluster Clinical Research Ethics Committee (CRE-2014.060) and the administration of the participating school.

### 2.2. Measures

#### 2.2.1. Observation System

The BEACHES system was used to code seven dimensions: (a) Child PA Level (lying down, sitting, standing, walking/moderate, or vigorous); (b) Child Location (inside home/dorm, outside home/dorm); (c) People Present (i.e., mother, father, other child, and other adult in the target child’s immediate environment); (d) Behavior Motivated (events related to motivating/influencing the target child to directly engage in PA or sedentary behavior, or to ingest food. These events could be antecedents (e.g., prompts) or consequences (e.g., praise)); (e) Motivator (none, mother, father, other child, and other adults); for example, a mother stating, “Go outside and play” would be coded as “Motivates PA, Mother”; (f) Views Media (whether or not the child engaged in media-related activity such as TV viewing); and (g) Eats (whether or not the child ingested food).

Child Activity Level, Location, and People Present were scored using momentary time sampling (i.e., codes were entered to score the occurrence each of these variables precisely at the end of each observation interval). Behavior Motivated, Motivator, Views Media, and Eats were scored using partial-interval time sampling (i.e., codes were entered if the event occurred at any time during each observation interval). Voice prompts from a digital recording paced alternating 15-s observe/record intervals (observers focused on the target child for 15-s and then had up to 15-s to enter codes onto a prepared recording form). During each cycle, one code per category was entered except for the People Present which was coded as ‘all that apply’.

#### 2.2.2. Observation Procedures

Observer training and maintenance. Data were collected by 20 research assistants trained using a standard protocol. They first memorized the operational definitions of the BEACHES behavioral dimensions and their subcategories and then learned the data recording procedures. Video examples and role-playing were used to demonstrate each category during classroom sessions; this was followed by both video and field practice observations. Training continued until an observer exceeded an inter-observer agreement (IOA) score of 85% on a criterion videotape (using interval-by-interval correspondence with agreements divided by agreements plus disagreements multiplied by 100). Additionally, during data collection approximately 10% of the total observations were scored independently and simultaneously by two data collectors. Results of these observations indicated good to excellent inter-observer reliability with a mean kappa ranging from 0.72 to 0.94 for all dimensions.

Observations at school. Each target child was observed during four unstructured sessions (i.e., before school, recess, lunch break, and after classes) during one normal school day per week over four consecutive weeks. Recess periods were for 30-min each, yielding 240 observation intervals per child (i.e., 60 intervals × 4 days). Lunch break observations were also for 30-min each, and began immediately after the child finished eating (i.e., yielding 240 observation intervals per child). The before school observations were scheduled to be initiated 30-min before the official start of the school day (240 observation intervals/child) and the after-class observations were planned for 60-min after the official end of school (480 observation intervals/per child). The actual length of the before school and after classes observation periods, however, depended on each child’s personal schedule and school timetable (e.g., availability of an after-school program on the day of observation).

Observations at home. The target children were observed at their home/residence (boarders were observed in their residential areas) on the same four days they observed at school. Observation periods were 60 min in length, and were conducted between the time the child arrived at the residence and before dinner started (yield = 480 observation intervals/child). Previous studies suggest that four 1-h observations can provide a reasonably stable estimate of child’s activity behavior at home [23].

All observations were done during standard natural conditions, and all people involved were asked to behave as usual. They were not informed of the exact variables of interest until the entire observation period was completed.

### 2.3. Data Analysis

To facilitate comparisons across settings in which observation periods were of different lengths, data were analyzed primarily in terms of proportion of intervals (or % time). Descriptive statistics included the mean proportion of observation intervals (or time) recorded for the PA categories and the environmental characteristics observed in the school and home settings. PA levels were summarized in order to report sedentary behavior (SB, the sum of the lying down, sitting, and standing codes) and moderate-to-vigorous PA (MVPA, the sum of the walking and vigorous codes).

Dependent variables were child MVPA, and independent variables included personal factors, such as age, BMI, gender (boys, girls), mobility (walked with mobility aid or not), grade level (primary, junior, and senior secondary levels), and physical and social contextual factors. Partial correlation was used to assess the relationship between MVPA and environmental variables while adjusting for age, body mass index (BMI), and mobility (i.e., walking with or without mobility aid) as confounders. To control for the effects of demographic variables (e.g., age, BMI, gender, mobility etc.), hierarchical multiple regression was used to examine the association of contextual variables with MVPA at each setting. Alpha level was set at *p* < 0.05 for all statistical tests.

## 3. Results

### 3.1. Characteristics of Participants and Their Background

Table 1 describes the participating children and their family backgrounds. Most participants were boys (74.3%), lived in residence at school during weekdays (77.1%), and were able to walk independently. Twenty-one were diagnosed as having cerebral palsy, and about one-third of the participants had intellectual disabilities. About 10% of the children’s parents had a college degree and about half the fathers and mothers reported middle or high school to be their highest educational level. In comparison to the Hong Kong median monthly household income of HK$24,900 (www.news.gov.hk/en/categories/finance/html/2017/06/20170609_164740.shtml), about half the families (52%) reported a monthly household income of less than HK$15,000.

### 3.2. Descriptive Statistics of Physical Activity and Contextual Factors

Table 2 presents the mean proportion of intervals (and calculated minutes) children spent in various activity levels as well the proportion and amount of time they spent indoors/outdoors and while alone or with other people in the school and home settings. Overall, the children were sedentary most of the time (ranging from 86.8% to 92.6% by setting) and primarily sitting down. They spent very little time engaged in MVPA (ranging from 7.3% to 13.2%), especially in vigorous PA (ranging from 0.7% to 3.7%). At school, they spent most of the observed time outdoors (about 98%) and at home spent 37% of their time there. Children were rarely alone, especially at school (only about 0.2% of the intervals) where they typically shared space with another child (about 94% of the time) and an adult such as teacher or playground supervisor (about 90% of the time). At home/in residence they were alone only about 7% of the time, typically sharing space with an adult who was not their parent (76% of the time), another child (66%), mother (18%), and father (8%).

Table 2 also identifies the proportion of intervals during which (a) selected behaviors were motivated; (b) who did the motivating; (c) media were viewed; and (d) food was ingested. At school, none of the specified behaviors were motivated (i.e., prompted or reinforced) during most (77%) of the observed intervals and at home none of them were motivated during about 80% of the intervals. Most motivating for the targeted behaviors came from an adult who was not the child’s parent. The school setting that had the greatest proportion of intervals in which PA engagement was promoted was after classes (19.3% of intervals); nonetheless, this same setting had the highest rate of intervals (11.9%) with prompts for engaging in sedentary behavior. At home, there were nearly twice as many intervals with prompts to engage in PA than to be sedentary (8.5% vs. 4.6%). The proportion of intervals in which students engaged in viewing electronic media at school ranged from 2% (before school) to 27.8% (after classes). Meanwhile, at home/in residence, children viewed electronic media during about 56% of the observed intervals. Prompts related to eating and viewing media were rare in all five settings.

### 3.3. Associations of Contextual Characteristics with Physical Activity

Table 3 shows the correlations between contextual variables and the proportion of time in MVPA across settings. At school, MVPA% was positively associated with prompts for PA at recess (*r* = 0.55), lunch break (*r* = 0.63), and after class (*r* = 0.49) periods; as well as with ‘other child’ (*r* = 0.56) as motivator during recess and with ‘other child’ (*r* = 0.58) and ‘mother’ (*r* = 0.65) during lunch break. As expected, MVPA% was negatively associated with viewing media (*r* = −0.36) during the after-class period. At home, MVPA% was positively associated with the presence of father (*r* = 0.75) and with ‘father’ (*r* = 0.77) as a motivator.

### 3.4. Predictors of Physical Activity at School and Home

Demographic factors such as age, BMI, gender, mobility, grade level, and residential boarding were entered at Step 1. Overall, only level of mobility was statistically significant, with children who walked without assistance engaging in more MVPA% during the recess (*p* < 0.001), lunch (*p* < 0.001), and after class (*p* = 0.001) periods than those who needed assistance. After entering the significant BEACHES variables at Step 2, similar results found that mobility was statistically significant at recess (*p* < 0.001), lunch (*p* < 0.001), and after class (*p* < 0.01) periods. Gender was found to be statistically significant after classes (*p* = 0.01), with boys having higher MVPA% than girls.

Specifically, at recess, demographic factors explained 58.6% of the variance in MVPA% at Step 1. After entering the significant BEACHES variables (i.e., prompts for active behavior from others and the presence of ‘other child’ motivator) at Step 2, the total variance explained by the model was 77.6%, F (8, 26) = 11.24, *p* < 0.001. These two BEACHES variables explained an additional 18.9% of the variance in MVPA%, after controlling for demographic variables, R squared change = 0.189, F change (2, 26) = 10.96, *p* < 0.001. Prompts from others to be active (β = 0.339, 95% CI = [0.123, 0.556], *p* < 0.01) and presence of ‘other child’ motivator (β= 0.288, 95% CI = [0.055, 0.521], *p* < 0.05) were significant predictors. At lunchtime, demographic factors explained 60.1% of the variance in MVPA% at Step 1. After entering the significant BEACHES variables (i.e., prompts for active behavior from others, presence of mother motivator, and presence of other child motivator) at Step 2, the total variance explained was 79.3%, F (9, 25) = 10.64, *p* < 0.001. These three BEACHES variables explained an additional 19.2% of the variance in MVPA%, R squared change = 0.192, F change (3, 25) = 7.71, *p* = 0.001, but they were not statistically significant in the final model. During the after-classes period, demographic factors explained 39.4% of the variance in MVPA%. After entering the significant BEACHES variables (i.e., prompts for active behavior from others and viewing media) at Step 2, the total variance explained was 62.5%, F (8, 26) = 5.42, *p* < 0.001. These two BEACHES variables explained an additional 23.1% of the variance in MVPA%, R squared change = 0.231, F change (2, 26) = 8.00, *p* < 0.01; with viewing media (β = −0.458, 95% CI = [−0.872, −0.044], *p* < 0.05) as a significant predictor.

At home, demographic factors explained 16.9% of the variance in MVPA%. After entering the significant BEACHES variables (i.e., presence of father and presence of father motivator) at Step 2, the total variance explained was 72.9%, F (9, 25) = 7.47, *p* < 0.001. These two BEACHES variables explained an additional 56% of the variance in MVPA%, R squared change = 0.560, F change (2, 25 = 25.82, *p* < 0.001). Presence of father (β= 0.558, 95% CI = [0.216, 0.900], *p* < 0.01), and the father being a motivator (β= 0.509, 95% CI = [0.173, 0.845], *p* < 0.01) were significant predictors.

## 4. Discussion

As an extension of our previous work using direct observation to document children’s PA, the present study is the first to assess activity levels and associated contextual characteristics of children with PD in both school and home settings. Consistent with previous studies [4,17], these children with PD were generally inactive in all settings, with school recess and lunch breaks providing slightly more PA accrual. Contextual factors such as presence of another child or adult, the absence of prompts for the targeted behaviors, and not ingesting food were commonplace across all settings. As expected, the children spent more time indoors and viewing media at home than at school. It is common for children with PD in Hong Kong to go home immediately after school to do homework and/or watch television before dinner; as well, small living spaces and inadequate play facilities typically restrict their active play opportunities [20].

The results of the partial correlations showed significant associations between children’s PA and several contextual variables at school and home. MVPA% was positively associated with prompts for PA across school settings and with peers as a motivator during recess and lunch breaks, thereby supporting the important role of social influences in motivating active behavior in children [21,24]. Interestingly, MVPA% was also positively related to mothers being motivators during lunch breaks. In many Hong Kong primary and special schools, parents (usually mothers) sometimes serve as volunteer monitors during lunchtime and they frequently bring lunchboxes to their children from home. Students have free time after lunch, and in the present study mothers were observed to encourage children to play when outside. Another interesting finding was that MVPA% at home was positively related to the presence of fathers and behaviors being motivation by them. The impact of fathers influencing children’s PA has been supported in previous studies [25,26], suggesting that fathers tend to engage in physical interactions with their child [27].

Results of the hierarchical multiple regression indicated that being able to walk without assistance was a significant demographic variable, confirming that physical function is a strong correlate of PA in children with PD [11]. Similar to the correlation findings, social support from both peers and parents were significant predictors of the children’s PA, a finding consistent with previous studies that examined children with PD [28,29,30]. These findings were in line with the ICF model, which suggests that PA is affected by interactions among various ICF components including body function (level of mobility), internal factors (personal demographic factors), and external factors (environmental conditions such as behavior being prompted by others).

Our study has several limitations. Although observers were trained to reduce potential reactivity, the effects of their presence on the children’s behavior, including PA, is unknown. Observations included both commuter and residential students in a wide range of grades from a single special school. On weekdays, ‘home’ for most of the 35 children (77%) was the school residential facilities and it was not possible to complete observations in their households when they returned on weekends. Thus, we were unable to determine the influence of parents on the PA of children who resided at the school. The school was designed specifically to educate children with PD, and thus extension of these findings to children with PD in mainstreamed schools (the case in many countries) and to other special schools is limited. Additionally, the limited sample size and insufficient variability for many variables prevented additional meaningful statistical analyses to be conducted.

## 5. Conclusions

This is the first study to document children’s PA and associated contextual characteristics at school (other than during recess) and at home/residence. Overall, the children accrued little MVPA either at school (only during about 11% of the time in the four school settings) and at home (only 7.4% of observed time). Thus, there is substantial need to provide children with PD, especially those unable to walk unassisted, with additional opportunities for PA throughout the day. Additionally, there were very few prompts for the children to engage in PA in any setting, thus educating parents and other childcare providers on the importance of PA and how to motivate it may prove relevant. Future studies should consider including: (a) larger sample of children and more schools; (b) assessments of behavior and contexts on weekends; and (c) more observations in the child’s actual residence and when both parents are present.

## Figures and Tables

**Table 1:** Characteristics of children with PD (N = 35) and their parents and families.

Variable	Category	Number	Percentage (%)
Gender	Male	26	74.3
	Female	9	25.7
Grade level	4–6	10	28.6
	7–9	11	31.4
	10–12	14	40.0
Mobility	Walked without assistance	19	54.3
	Walked with assistance	16	45.7
Boarding	Yes	27	77.1
	No	8	22.9
Type of PD	Cerebral Palsy	21	60.0
	Other types	14	40.0
Education (highest)			
Father	Elementary	7	20.0
	Middle or high school	17	48.6
	College or above	11	31.4
Mother	Elementary	4	11.4
	Middle or high school	21	60.0
	College or above	10	28.6
Employment status			
Father	Self-employed	8	22.9
	Full-time employed	18	51.4
	Part-time employed	4	11.4
	Unemployed	5	14.3
Mother	Self-employed	3	8.6
	Full-time employed	9	25.7
	Part-time employed	7	20.0
	Unemployed	16	45.7
Household monthly income	<$8000	3	8.6
	$8000–15,000	15	42.9
	$15,001–24,000	7	20.0
	$24,001–50,000	3	8.5
	>$50,001	7	20.0

**Table 2:** Child physical activity levels and contextual characteristics at school and home.

Category	School				Home
	Before School	Recess	Lunch Break	After Classes	Before Dinner
	Mean Min. = 14.5	Mean Min. = 26.3	Mean Min. = 29.6	Mean Min. = 52.4	Mean Min. = 42.0
*Activity level*					
Lying down % (min)	3.2 (0.5)	1.4 (0.4)	1.0 (0.3)	0.3 (0.2)	2.0 (0.8)
Sitting % (min)	74.3 (10.8)	65.4 (17.2)	63.7 (18.8)	67.1 (35.1)	75.2 (31.6)
Standing % (min)	15.2 (2.2)	20.4 (5.4)	22.1 (6.5)	21.1 (11.0)	15.5 (6.5)
Walking % (min)	5.4 (0.8)	10.6 (2.8)	10.9 (3.2)	7.9 (4.1)	6.8 (2.8)
Vigorous % (min)	1.9 (0.3)	2.1 (0.6)	2.3 (0.7)	3.7 (1.9)	0.7 (0.3)
Sedentary **^a^** % (min)	92.7 (13.4)	87.3 (23.0)	86.8 (25.7)	88.5 (46.3)	92.6 (38.9)
MVPA **^b^** % (min)	7.3 (1.1)	12.7 (3.3)	13.2 (3.9)	11.5 (6.0)	7.4 (3.1)
*Location*					
Inside % (min)	0.0 (0.0)	0.0 (0.0)	0.0 (0.0)	4.5 (2.4)	62.6 (26.3)
Outside % (min)	100.0 (14.5)	100.0 (26.3)	100.0 (29.6)	95.5 (50.0)	37.4 (15.7)
*People there*					
Alone % (min)	0.2 (0.0)	1.8 (0.5)	2.3 (0.7)	2.0 (1.4)	7.3 (3.1)
Mother % (min)	0.0 (0.0)	0.6 (0.1)	0.6 (0.2)	0.0 (0.0)	18.2 (7.6)
Father % (min)	0.0 (0.0)	0.1 (0.0)	0.6 (0.2)	0.0 (0.0)	7.5 (3.2)
Other child % (min)	98.8 (14.3)	94.1 (24.7)	94.4 (27.9)	89.8 (47.0)	65.7 (27.6)
Other adult % (min)	96.4 (14.0)	86.6 (22.8)	80.5 (23.8)	94.8 (49.6)	75.8 (31.9)
*Behavior motivated* **^†^**					
None %	87.1	76.6	79.8	65.3	79.6
PA %	7.7	7.7	6.5	19.3	8.5
SB %	4.5	10.1	8.9	11.9	4.6
Eating %	0.0	3.5	2.6	0.2	1.7
Not eating %	0.0	0.1	0.0	0.0	0.5
Media %	0.6	1.9	2.1	3.3	4.9
No media %	0.1	0.1	0.2	0.0	0.2
*Motivator* **^†^**					
None %	87.1	76.7	79.8	65.1	79.9
Mother %	0.0	0.5	0.3	0.0	2.9
Father %	0.0	0.0	0.0	0.0	0.2
Other child %	0.1	6.4	5.5	1.9	4.7
Other adult %	12.8	16.3	14.5	33.0	12.3
*Viewing media* **^†^**					
Yes %	2.0	12.3	16.1	27.8	56.3
No %	98.0	87.7	83.9	72.3	43.7
*Eating behavior* **^†^**					
Yes %	0.0	5.2	4.4	0.5	2.2
No %	100.0	94.8	95.6	99.6	97.8

**^a^** Sedentary = lying down + sitting + standing; **^b^** MVPA = walking + vigorous. PA = physical activity, SB = sedentary behavior, NE = no eating, NM = no media. NOTE: Time cannot be estimated for categories; **^†^** when using partial interval recording.

**Table 3:** Partial correlations between contextual characteristics and the percentage of time spent in moderate-to-vigorous PA (MVPA%) controlling for age, body mass index, and mobility.

Category	School			Home
	Recess	Lunch Break	After Classes	Before Dinner
*Location*				
Inside %	^a^	^a^	0.00	0.03
Outside %	^a^	^a^	0.00	−0.03
*People there*				
Alone %	0.04	0.12	−0.01	−0.14
Mother %	0.03	−0.26	^a^	0.34
Father %	0.31	−0.26	^a^	0.75 **
Other child %	−0.07	−0.11	0.07	−0.22
Other adult %	−0.19	−0.32	0.04	−0.28
*Behavior motivated*				
None motivated %	−0.27	−0.21	−0.29	0.01
PA motivated %	0.55 **	0.63 **	0.49 **	0.05
SB motivated %	0.11	−0.16	−0.04	0.04
E motivated %	−0.17	−0.15	−0.00	−0.09
NE motivated %	−0.28	0.07	−0.10	−0.18
M motivated %	−0.05	0.04	−0.34	−0.05
NM motivated %	−0.04	−0.06	−0.03	0.01
*Motivator*				
None %	−0.28	−0.21	−0.29	0.00
Mother %	0.03	0.65 **	^a^	0.24
Father %	^a^	^a^	^a^	0.77 **
Other child %	0.56 **	0.58 **	0.32	−0.05
Other adult %	−0.02	−0.22	0.24	−0.10
*Viewing media*				
Yes %	−0.04	−0.02	−0.36 *	0.14
No %	0.04	0.02	0.36 *	−0.14
*Eating behavior*				
Yes %	−0.24	−0.17	0.11	−0.09
No %	0.24	0.17	−0.11	0.09

^a^ correlation coefficient cannot be computed because the contextual variable is constant (=0). PA = physical activity, SB = sedentary behavior, NE = no eating, NM = no media; * *p* < 0.05; ** *p* < 0.01.
